# A Systematic Review on Health Resilience to Economic Crises

**DOI:** 10.1371/journal.pone.0123117

**Published:** 2015-04-23

**Authors:** Ketevan Glonti, Vladimir S. Gordeev, Yevgeniy Goryakin, Aaron Reeves, David Stuckler, Martin McKee, Bayard Roberts

**Affiliations:** 1 ECOHOST—The Centre of Health and Social Change, London School of Hygiene and Tropical Medicine, London, United Kingdom; 2 Norwich Medical School, University of East Anglia, Norwich, United Kingdom; 3 Department of Sociology, Oxford University, Oxford, United Kingdom; National Center of Neurology and Psychiatry, JAPAN

## Abstract

**Background:**

The health effects of recent economic crises differ markedly by population group. The objective of this systematic review is to examine evidence from longitudinal studies on factors influencing resilience for any health outcome or health behaviour among the general population living in countries exposed to financial crises.

**Methods:**

We systematically reviewed studies from six electronic databases (EMBASE, Global Health, MEDLINE, PsycINFO, Scopus, Web of Science) which used quantitative longitudinal study designs and included: (i) exposure to an economic crisis; (ii) changes in health outcomes/behaviours over time; (iii) statistical tests of associations of health risk and/or protective factors with health outcomes/behaviours. The quality of the selected studies was appraised using the Quality Assessment Tool for Quantitative Studies. PRISMA reporting guidelines were followed.

**Results:**

From 14,584 retrieved records, 22 studies met the eligibility criteria. These studies were conducted across 10 countries in Asia, Europe and North America over the past two decades. Ten socio-demographic factors that increased or protected against health risk were identified: gender, age, education, marital status, household size, employment/occupation, income/ financial constraints, personal beliefs, health status, area of residence, and social relations. These studies addressed physical health, mortality, suicide and suicide attempts, mental health, and health behaviours. Women’s mental health appeared more susceptible to crises than men’s. Lower income levels were associated with greater increases in cardiovascular disease, mortality and worse mental health. Employment status was associated with changes in mental health. Associations with age, marital status, and education were less consistent, although higher education was associated with healthier behaviours.

**Conclusions:**

Despite widespread rhetoric about the importance of resilience, there was a dearth of studies which operationalised resilience factors. Future conceptual and empirical research is needed to develop the epidemiology of resilience.

## Introduction

Why are some people able to cope during economic crises while others are not? The worst economic crisis in Europe since the Great Depression has already seen marked rises in the prevalence of poor mental health [1,2,3,4]. Suicides have risen across Europe and North America [5], especially in the countries worst affected by the crisis [6,7,8] and within these countries, in the regions where job losses have been highest [9]. Many economies continue to stagnate while social safety nets are being eroded and job security is increasingly precarious [10,11]. Yet not everyone exposed to the consequences of economic crisis, such as job loss, bankruptcy, or home repossession, is equally affected. Some individuals display a high degree of resilience while others seem especially vulnerable [12].

Resilience can be be defined as a dynamic process by which individuals, communities and societies positively adapt to significant adversity [13]. In light of the recent financial crisis, the value of resilience as a concept has been highlighted by health policy-makers and rearchers [14]. Thus, elucidating risk and protective factors that influence resilience during economic crises could help inform policy responses to mitigate negative health effects of economic crises. There is considerable research on resilience in disciplines such as individual traits in psychiatry, social relationships in sociology, family relationships in psychology, responses to violence and humanitarian crises, and systems level responses to violence, humanitarian crises, and pandemic disease outbreak [13,15,16]. However, the evidence on health resilience and its risk and protective factors in the context of economic crises remains unclear. As a result, the potential value of resilience for developing policies to protect against the effects of economic shocks such as those that characterize the current period of austerity is unclear. The objective of this systematic review is to examine evidence from longitudinal studies on factors influencing resilience for any health outcome or health behaviour among the general population living in countries exposed to financial crises.

## Methods

We conducted a systematic review of published primary research that investigated factors related to resilience to the negative health consequences of economic crises.

### Inclusion criteria

Studies were included if they examined: (i) exposure to a specific economic crisis; (ii) changes in health outcomes or behaviours over time; and (iii) statistical data on associations of health risk and/or protective factors with health outcomes/behaviours. There were no time period restrictions. The publication language was restricted to English only. We only considered primary research studies which used quantitative longitudinal designs (panel/cohort and repeated cross-sectional) that capture changes in these factors over time given the temporal, dynamic nature of resilience. The studies therefore had to have at least two time points of observation (i.e. pre- and/or during, and post-crisis periods).

We identified economic crises as those meeting the criteria of “a significant decline in economic activity spread across the economy, lasting more than a few months, normally visible in real GDP, real income, employment, industrial production, and wholesale-retail sales. A recession begins just after the economy reaches a peak of activity and ends as economy reaches its trough”[17]. We considered a risk and/or protective factor as any attribute, characteristic or exposure of an individual that increases or decreases the likelihood of developing a disease or injury [14].

### Search strategy

Six bibliographic databases (EMBASE, Global Health, MEDLINE, PsycINFO, Scopus, Web of Science) were searched between July and October 2013. The broad and varied terminology around resilience and related concepts of vulnerability, risk and protective factors meant that we did not use such terms in our search in order to maximise sensitivity. To capture a broad range of effects associated with economic crises we included the following search terms: economic shock, economic recession, recession, economic crisis, financial crisis, fiscal crisis, banking crisis, economic depression, economic hardship, economic insecurity, austerity, financial constraint, economic downturn, economic change, economic breakdown, economic turmoil, economic stagnation, economic adversity, economic turbulence, and macroeconomic fluctuation. In addition, references of all the final included papers were searched for articles not identified through electronic searches. A full description of the search terms, strategy and screening stages can be found in S1 Table.

### Study selection, data extraction and analysis

Results from the bibliographic databases were merged and duplicates were removed. Two reviewers (KG and VG) independently screened the search results by title, abstract and full text. Disagreements were resolved by discussion and consensus.

We extracted the following information from the studies included in the review: country, economic crisis and years examined, study population, study design, methods of analysis, risk and protective factors, health outcome(s) and health behavioural outcome(s). Relevant information from retrieved articles was extracted for a narrative synthesis by both reviewers and summarized in Table 1. Narrative synthesis was used due to heterogeneity in study designs, methods, risk/protective factors, health outcomes, presentation of data and study contexts. In addition, some of the results relevant to this review were only secondary objectives of the included papers, which made data extraction and interpretation more complex. For this reason we did not extract the effect sizes presented in the original studies.

This review follows the Preferred Reporting Items for Systematic Reviews and Meta-Analyses [18] (S1 PRISMA Checklist).

**Table 1:** Overview of included studies.

Source	Country	Economic crisis and year studied	Study design	Analysis	Health outcome(s)	Health behavioural outcome(s)	Risk/protective factor
Agudelo-Suarez, A.A. et al. 2011 [33]	Spain	2008 economic crisis; (2008; 2011)	Cohort	Descriptive analysis with CI; multivariate logistic regression with random effects	Mental health	N/A	Gender; Employment/occupation; Income/financial constraint; Social relations
Astell-Burt, T. et al. 2013 [36]	United Kingdom	2008 economic crisis; (2006–2010)	Repeated cross-sectional	Multivariate logistic regression	Depression, mental illness, cardiovascular and respiratory illness	N/A	Employment/occupation
Bor, J. et al. 2013 [38]	USA	2008 economic crisis; (2006–2010)	Repeated cross-sectional	Weighed multivariate OLS regression	N/A	Self-reported alcohol use	Gender; Age; Education; Marital status; Employment/occupation; Income/financial constraint
Doherty, A. M. et al. 2013 [28]	Ireland	2007–2008 economic crisis; (2003; 2005; 2007; 2009)	Repeated cross-sectional	Multivariable linear regression	Self-rated happiness	N/A	Age; Personal beliefs
Dregan, A. et al. 2009 [37]	United Kingdom	1990s economic crisis; (1984–1985; 1991–1992; 1998; 1999; 2001; 2002–2003; 2004–2005; 2006–2007)	Repeated cross-sectional	Multivariate regression	Sleep disturbance	N/A	Gender; Age; Income/financial constraint; Mental health
Economou, M. et al. 2013 [24]	Greece	2008 economic crisis; (2008; 2011)	Repeated cross-sectional	Multivariable logistic regression	Depression	N/A	Gender; Marital status/household size
Gili, M. et al. 2012 [1]	Spain	2007 economic crisis; (2006; 2010)	Cohort	Multivariate logistic regression	Major and minor depression disorder, dysthymia,	Alcohol abuse	Gender; Education; Income/financial constraint
Gudmundsdottir, D.G. et al. 2013 [25]	Iceland	2007 economic crisis; (2007; 2009)	Cohort	Paired t-test; multiple linear regression	Happiness	N/A	Age; Marital status/household size; Income/financial constraint; Health status; Social relations
Hauksdottir, A. et al. 2013 [26]	Iceland	2008 economic crisis; (2007–2009)	Cohort	Binary logistic regression	Perceived stress	N/A	Gender; Age; Education; Marital status/household size; Employment/occupation; Income/financial constraint; Area of residence
Katikireddi, S. V. et al. 2012 [2]	England	2008 economic crisis; (1991–2010)	Repeated cross-sectional	Poisson regression, logistic regression	Mental health	N/A	Gender; Age; Education; Employment/occupation; Income/financial constraint; Area of residence
Kim, I-H. et al. 2011 [31]	South Korea	1997 economic crisis; (1995; 1999; 2003; 2006)	Repeated cross-sectional	Prevalence ratios, log-binominal regressions; time trends	Self-perceived health	N/A	Employment/occupation
Kondo, N. et al. 2008 [29]	Japan	1997–1998 Asian economic crisis; (1986; 1989; 1998; 2001)	Repeated cross-sectional	Logistic regression; multivariate generalised estimating equations with a logit link function;	Self-perceived health	N/A	Employment/occupation
Lammintausta, A. et al. 2012 [20]	Finland	1990s economic crisis; (1993–2002 (5-year intervals))	Register-based data	Poisson regression	Incidence of coronary heart diseases (CHD) and mortality	N/A	Gender; Income/financial constraint
Lee, W. Y. et al. 2009 [32]	South Korea	2007 economic crisis; (1993–2006 (5-year intervals))	Register-based data	Poisson regression, relative index of inequality, slope of index of inequality	Suicides	N/A	Education
Macy, J. T. et al. 2013 [39]	USA	2008 economic crisis; (1987; 1993; 1999; 2005; 2009; 2011)	Cohort	Bivariate correlations, hierarchical multiple regression, logistic regression	N/A	Healthy eating: check ingredient label when buying food, choose food to eat based on health value. Sporting: frequency of vigorous exercise. Smoking. Wearing safety belt.	Gender; Age; Education; Marital status/household size; Income/financial contraint
Martikainen, P. et al. 2007 [21]	Finland	1990s economic crisis; (1989; 1994)	Cohort	Cox regression	Mortality	N/A	Employment/occupation
McClure, C. B. et al. 2012 [27]	Iceland	2008 economic crisis; (2007; 2009)	Cohort	Binary logistic regression	N/A	Smoking (relapse and cessation)	Gender; Age; Education; Employment/occupation
Montgomery, S. et al. 2013 [34]	Sweden	1990s economic crisis; (1990; 2001)	Cohort	Cox regression	All-cause mortality	N/A	Employment/occupation
Ostamo, A. et al. 2001 [22]	Finland	1990s economic crisis; (1989–90; 1991–1996; 1997)	Cohort	Chi-square test	Attempted suicide	N/A	Gender; Age; Income/financial constraint; Health status
Rahmqvist, M. et al. 1998 [35]	Sweden	1990s economic crisis; (1989–1995)	Repeated cross-sectional	Multivariate logistic regression	Psychological distress (self-reported symptoms of anxiety, anguish, depression, and sleeplessness)	N/A	Gender; Employment/occupation
Viinamaki et al. 2000 [23]	Finland	1990s economic crisis; (1993–1995)	Repeated cross-sectional	Chi-square and t-tests; Mann-whitner U test; logistic regression	Mental well-being	N/A	Gender; Age; Employment/occupation; Income/financial contraint; Personal beliefs; Health status; Social relations
Wada, K. et al. 2012 [30]	Japan	1997–99 Asian economic crisis; (1980–2005)	Register-based data	Generalized estimating equation model	Mortality, suicide	N/A	Employment/occupation

### Assessment of the methodological quality

We used an adapted version of the Quality Assessment Tool for Quantitative Studies (developed by the Effective Public Health Practice Project [19]) to assess the methodological quality of the included studies (S1 Quality Appraisal). The tool contains 19 items in eight key domains: (1) study design; (2) blinding; (3) representativeness in the sense of selection bias; (4) representativeness in the sense of withdrawals/drop outs; (5) confounders; (6) data collection; (7) data analysis; and (8) reporting. Studies can have between six and eight component ratings, with each component score ranging from 1 (low risk-of bias; high methodological quality) to 3 (high risk-of-bias; low methodological quality). An overall rating for each study was determined based on the component ratings. For example, if eight ratings have been given, a rating of ‘*strong’* was attributed to those with no weak ratings and at least five strong ratings, ‘*moderate’ to* those with one weak rating or fewer than five strong ratings, ‘*weak’* attributed to those with two or more weak ratings. To minimize the risk of bias, assessments were completed independently by two reviewers (KG and VG). The ratings for each of the eight domains, as well as the total rating, were compared and consensus reached on a final rating for each included article.

## Results

**Fig 1:**
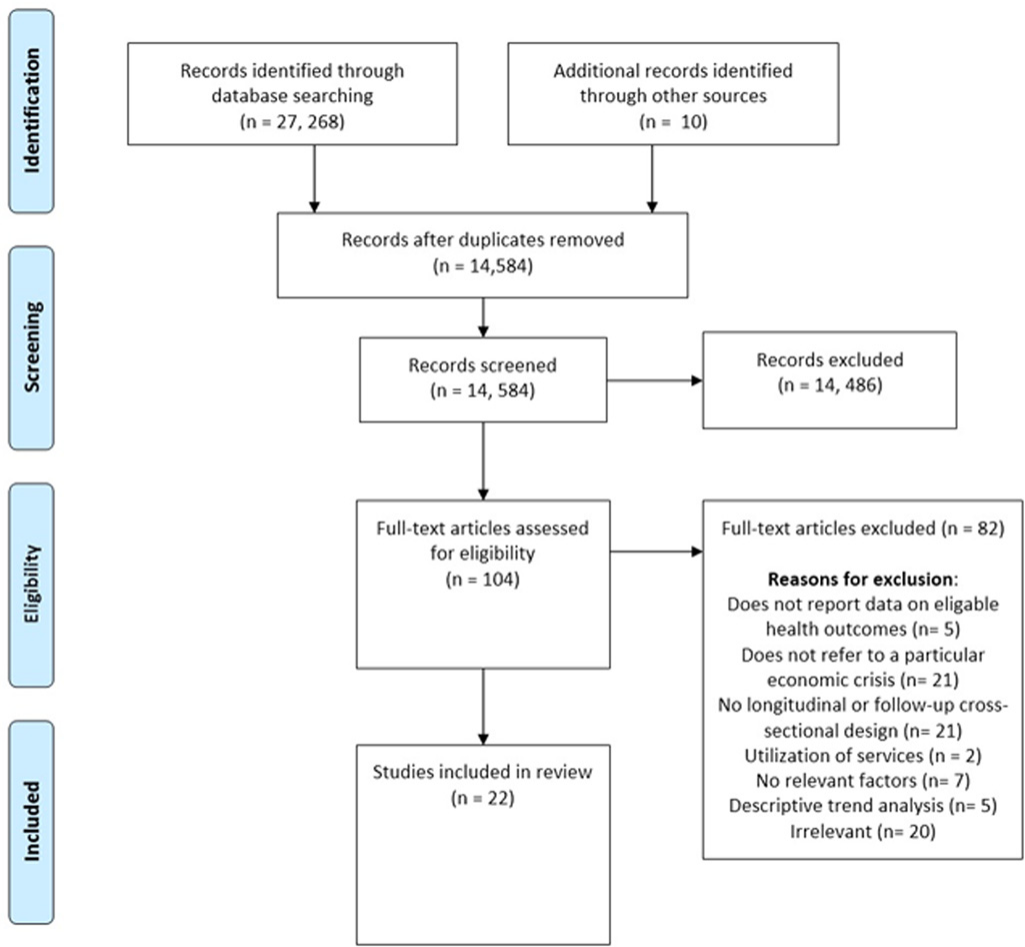
Results of screening process.

The results of the screening process are shown in Fig 1. In total, 14,584 articles were screened by title and abstract for possible inclusion in the review. The full text of 104 articles were obtained and assessed for eligibility. Twenty two studies met the eligibility criteria and were included in the final review. The main reasons for exclusion were that papers: contained descriptive analyses only; reported macro-level trends without reporting on individual or household characteristics; were cross-sectional studies of a single point in time; and focused solely on health service utilization and performance.

The 22 included studies were conducted in 10 countries: Finland [20,21,22,23], Greece [24], Iceland [25,26,27], Ireland [28], Japan [29,30], South Korea [31,32], Spain [1,33], Sweden [34,35], the United Kingdom (UK) [2,36,37], and the United States of America (USA) [38,39]. Studies examined the following economic crises: the early 1990 recession in the UK (1991–1992), the Asian economic crisis (1997–1999), the Finnish Banking Crisis (1991–1993), the Swedish Banking Crisis (1991–1993) and the Global Economic Crisis, which encompasses the Irish financial crisis (2007–2008), US Great Recession (2007–2008), the Icelandic financial crisis (2008–2011), the UK financial crisis (which started in 2008), the Greek financial crisis (which started in 2008) and the Spanish financial crisis (which started in 2008).

Of the 22 selected studies, nine were cohort studies [1,21,22,25,26,27,33,39,40], three were register-based cohort studies (20, 30, 32) and ten were repeated cross-sectional studies [2,23,24,28,29,31,35,36,37,38]. Study characteristics are shown in Table 1.

We identified the following 10 broad risk/protective factors associated with health during economic crises: gender, age, education, marital status and household size, employment/occupation, income/ financial constraints, personal beliefs, health status, area of residence, and social relations. The findings related to these 10 factors are described below and in Table 2, using sub-categories of the following main types of health outcomes associated with the risk and/or protective factors: physical health, mortality, suicide (including attempted), mental health, and health behaviours.

**Table 2:** Summary of risk (-) and protective (+) factors for health outcomes and behaviours during economic crises (studies in parentheses).

		Physical health	Mortality	Suicide/attempt-ed suicide	Mental health						Health behaviour			
					General mental health	Psychological distress^1^	Stress	Depression^2^	Sleep quality	Happiness	Alcohol consumption^3^	Binge drinking	Smoking^4^	Engaging in healthy behaviours^5^
**Gender**	Male				- [33];- [2]	- [35]		+ [1]			- [1]	- [38]		
	Female	- [29]			- [23]		- [26]	- [24]	- [37]				+ [27]	+ [39]
**Age group**	Young^6^			- [22]			- [26]							
	Middle-age^7^	- [29]					- [26]	- [24]				+ [38]	+ [27]	
	Elderly^8^						- [26]	- [24]		+ [25]		+ [38]	+ [27]	
	Increasing age	+ [23]						+ [24]	+ [37]	+ [28]			- [39]	
**Education**	Low^9^			- [32]			+ [26]							
	Middle^10^						- [26]	+ [1];- [24]					+ [27]	
	High^11^						+ [26]	+ [1]			+ [1]	+ (38)	+ [27]	+ [39]
**Marital status and children in household**	Single							- [24]						
	Unmarried	- [29]										- [38]		
	In partnership									+ [25]				
	Married /cohabiting						- [26]	- [24]		+ [25]				+ [39]
	Divorced	+ [29]												
	Children in household size^12^						- [26]					+ [38]		
**Employ-ment**	Employed					- [26]		- [24]						
	Unemployed/Home maker	− [29];− [36]	− [21];− [34]		- [33];- [36]	- [1];- [35]		− [1];− [36];− [24]		- [25]	- [1]	+ [38]		
	Unable to work/ disability											+ [38]		
	Temporary/ Daily^13^	- [31]												
	Student					- [26]						+ [38]		
	Retired								+ [37]			+ [38]		
	Changes in employment status											- [38]		
**Occupation**	Non-professional/Manual	− [31]; − [29]												
	Professional/Managerial		- [30]	- [30]										
**Income**	Low^14^		- [20]		- [29];- [33]									
	Middle^15^						- [26]					- [38]		
	High													
	Financial constraint^16^							- [24];- [1]	- [37]	- [25]				- [39]
	Income rise												- [27]	
	Income drop												+ [27]	
**Personal beliefs**	Lack of confidence in the future				- [23]									
	Spiritual belief^17^									+ [28]				
**Health status**	Good perceived health									+ [25]				
	Poor perceived health				- [23]				- [37]					
**Area of residence**	Farming community						+ [26]							
**Social relations**	Having financially dependent relatives				- [33]									
	Problems with partner				- [23]									
	Satisfactory social relationships									+ [25]				

**+ Is a protective factor**

**- Is a risk factor**

**See text for further information on reference categories for risk/protective factors**

**Table notes:**

^1^ Including: generalised anxiety and multi-somatoform disorders

^2^ Including: depressive disorders and dysthymia

^3^ Alcohol abuse

^4^ Quit rate

^5^ Including: check food ingredient labels, choosing food based on health values, increased frequency of vigorous exercise, wearing seatbelt

^6^ Based on different age group definitions in the papers, young age was defined as 15–24 years old

^7^ Based on different age group definitions in the papers, middle age was defined as middle age 25–60 years old

^8^ Based on different age group definitions in the papers, elderly was defined as 60+ years old

^9^ Including: basic education: grades 1–6, primary school, no schooling

^10^ Including: grades 7–12

^11^ University, college/graduate school

^12^ Includes categories of no children in the household, and children aged <18 in the household

^13^ Limited contractual work of less than one year or daily contractual work of duration of one month or less

^14^ Including: low income tertiles (€ 9000, € 13,000 and € 20,000), low salary (≤ € 1,200)

^15^ Household income over US$ 25,000

^16^ Including: financial hardship: ‘difficult making ends meet’, mortage repayment difficulties and eviction

^17^ Including: community trust, religious belief, and satisfaction with democracy

### Methodological quality assessment

All of the 22 included studies used an observational study design. The quality assessment of the included studies found that 12 had a low risk of bias and 10 had a moderate risk of bias (full details are provided in S2 Table and S1 Fig). In terms of representativeness of the wider population, all of the studies were rated as ‘strong’ with the exception of one study that was rated as ‘moderate’. We were able to rate nine studies for representativeness relating to withdrawals and drop-outs: one study was rated as ‘weak’, five studies were rated as ‘moderate’ and three received a ‘strong’ rating. Eight studies received a ‘moderate’ rating for confounding, while 14 studies were at low risk of confounding. Four studies scored ‘weak’ on data collection as they did not provide any information about the validity and reliability of their measures; two of these studies used the GHQ questionnaire but did not provide any information on the specifics of the study population. The remaining 16 scored ‘moderate’ and two studies scored ‘strong’ for data collection. One study achieved a moderate score with regards to data analysis, and the remaining 21 studies received a strong rating. One study had a moderate score in reporting quality, while the remaining 22 studies showed high quality of reporting and received ‘strong’ ratings.

### Gender

#### Physical health, mortality and suicide (including attempted suicide)

Two studies looked for gender differences in physical health, mortality or suicide during recessions. A study by Ostamo et al. examined the association between the economic recession in Finland (1989–1997) and trends in attempted suicides treated in health facilities in Helsinki. The 9 year study indicated that Finland’s economic recession of the 1990s was not associated with increased attempted suicides, and that while suicide attempt rates among males remained higher than among females, they declined more than female rates during the study period [22]. Kondo et al. examined the impact of the Japanese economic recession (1991–2000) on perceived health status, finding that females were more likely than males to report poor self-rated health both before and after the recession, and they had increased probability of doing so following the crisis [29].

#### Mental health

We found evidence of an association between gender and general mental health status [1,2,23,33], stress levels [26], psychological distress [35], depression [24], and sleep quality [37]. The impact of economic recession on mental health in the early 1990s was examined in the population of three countries (UK, Finland, and Sweden) by Dregan et al., who found that the chance of experiencing sleep problems through worry (measured by lost sleep over worry items in the GHQ-30) among individuals aged ≥50 years was higher in females than males both before (1984) and during the recession in UK. However, there was only a marginal increase in sleep problems among females during the recession [37]. Viinamaki et al. recorded increasing levels of mental disorders (measured by GHQ-12) among both men and women during the economic recession of the early 1990s in Finland, with higher rates among females than males. In the post-crisis period (1995), the prevalence of mental disorders decreased in males but remained unchanged among females [23]. Similar results were reported by Rahmqvist et al. in their study in Sweden, with the 1991 recession (1991–1993) associated with increased prevalence of psychological distress in both genders relative to pre-crisis period (1989), but with the largest increase among males [35].

Gili et al. assessed mental health risks in the general Spanish population and found that males were less likely than females to experience major depression and dysthymia both before (2006) and during the crisis years (2010–2011), but the likelihood of experiencing depression increased for males during the crisis period as compared to the pre-crisis period [1]. Agudelo-Suarez et al. focused on the subgroup of Spanish migrant workers and their mental health (assessed by the GHQ-12) and noted that both genders experienced an increase in the prevalence of poor mental health during the crisis in 2011 (as compared to the pre-crisis period in 2008), but this was only significant for males [33]. Similar findings were reported in England, where Katikireddi et al. examined differences in population mental health (measured using GHQ-12) during and between two economic crises (early 1990s and in 2009–2010). The prevalence of poor mental health was consistently higher among females than males over most of the study period, but the increase in reporting poor mental health during both recessions compared with the base year (2008) was greater among males [2].

In Iceland, Hauksdottir et al. looked at changes in self-reported levels of stress as a result of the Icelandic economic crisis in 2008. The age-adjusted mean perceived stress levels (measured using the PSS-4 score) in 2009 increased significantly from 2007 levels among females but not males, leading to increased reporting of high stress levels in females but not males during the crisis in 2009 (as compared to the pre-crisis period in 2007) [26]. In Greece, Economou et al. described how during the crisis period (2011) the prevalence of major depression increased in both genders as compared to the pre-crisis period (2008), but it remained higher among females just as in the pre-crisis period (2008) [24].

#### Health behaviour

Four studies investigated changes in health behaviour by gender [1,27,38,39].

Bor et al. looked at alcohol consumption during the Great Recession 2008–2009 in the USA and found that males were more likely than females to engage in frequent binge-drinking during the crisis (2008–2009) but their probability of doing so was lower than before the crisis (2006–2007) [38].

Gili et al. found that in the general Spanish population males (as compared to females) were more likely to abuse alcohol (2010–2011), but to a lesser extent during the crisis than before it (2006) [1]. Macy et al. found that even before the US Great Recession (2005), females were more likely than males to check food ingredient labels but the difference widened during the crisis (2011). Being female was also associated with choosing food based on health values (a separate outcome variable) but this association did not change significantly from before to during the crisis period [39]. A study by McClure et al. found that the prevalence of smoking fell significantly among men and women following the 2008 economic crisis, but the quit rate was greater among females [27].

### Age

#### Physical health and suicide

Kondo et al. examined the health impact of the Japanese economic recession (1991–2000) comparing pre- and post-crisis periods (1986/1989 and 1998/2001) and showed those aged 40–60 (as compared to 20–39) were more likely to report poor health both before (1986/1989) and after-crisis years (1998/2001), but this likelihood was lower after the crisis compared to the pre-crisis period [29].

In their study of the Finnish economic recession (1989–1997), Ostamo et al. observed that although the overall rates of attempted suicide decreased significantly among both females and males over the whole study period, suicide attempts by 15 to 24-year-olds of both genders increased after 1994. Moreover, as the economic crisis unfolded, a clear decreasing trend was noted only among 25 to 34-year-old men [22].

#### Mental health

We found evidence of associations between age and changes in general mental health status [2,23], stress levels [26], depression [24], sleep disturbance [37], happiness [25,28], during economic crises.

The study by Katikireddi et al. during two economic recessions in England (early 1990s and in 2009–2010)) found that changes in mental health during the recession periods were not confined to any specific age group (25–35, 35–44, 45–54, 55–64) [2]. Viinamaki et al. found that during the recession in Finland (1993), increasing age was associated with better mental health (measured by the GHQ) but only in men and only during the recession (1993). Age was not an independent risk factor for impaired mental health, either among males or females, at the end of the crisis or in the post-crisis period (1994–1995) [23]. In a study of stress during the Icelandic crisis in 2009, Hauksdottir et al. found that the likelihood of experiencing high stress levels during the economic crisis (2009) was higher among females belonging to the 18–29, 40–49, and 60–69 age groups when compared to the same age groups in the pre-crisis period in 2007, but there was no such significant association with age for males [26].

Economou et al. found that substantial increases in the prevalence of depression were found in several age groups (<34, 25–34, 45–54, 55–64) during the Greek crisis (2011) as compared to the pre-crisis period (2008), and also that each year increase in age decreased the chances of manifesting major depression [24]. Similarly, a study by Dregan et al. conducted in the UK among individuals over ≥50, found that during the recession in 1991 sleep loss through worry declined with increasing age [37]. Further evidence on the role of age was found in studies that focused on happiness. Doherty et al. examined individual happiness before (2003–2007) and during (2009) the economic crisis in Ireland and found that the association between an increase in age (as a continuous measure) and greater happiness was stronger prior to the crisis (2003–2007) and then weakened during the crisis (2009) [28]. Gudmundsdottir observed that those aged 70–79 reported higher happiness levels (as compared to those aged 18–29 years) both before (2007) and during (2009) the Icelandic economics crisis [25].

#### Health behaviours

Age was also associated with health behaviours during economic crises [27,38,39]. Bor et al. found that those aged 30–49, 50–64, 65+ years (with 18–29 years as a reference group for all age groups) had lower chances of being frequent binge drinkers during the US Great Recession in 2008–2009. The authors also noted that there was a relative increase in the likelihood of frequent binging among persons aged 25–34 and 55–59 (as compared to 18–29 years old) during the crisis period (2008–2009) compared with the pre-crisis period (2006–2007) [38]. Macy et al. showed that in the US Great Recession, increased age was associated with smoking on daily basis in both pre-crisis (2005) and during crisis period (2011), but this association was weaker during the crisis period [39]. McClure et al. observed that former smokers in older age groups (aged 40–59 and ≥60+ vs. 18–39) had a lower chance of relapse during the Icelandic crisis period (2009) regardless of gender. During the crisis, women in the age group 30–39 were less likely to quit smoking compared to women aged 18–29 [27].

### Education

#### Suicide (including attempted suicide)

Lee et al. found that increases in suicides were concentrated among those with lower education during and after the economic crisis of 1997 in South Korea, in both genders and all age groups [32].

#### Mental health

Several studies examined associations between education level and changes in general mental health status [2], stress levels [26] and depression [1,24] during economic crises. Katikireddi et al. observed that adjustment for changes in educational level did not explain the increases in poor mental health among men or women during two economic recessions in England (in the early 1990s and in 2008 onwards) [2]. However, Hauksdottir et al. found that the likelihood of experiencing high stress levels during times of crisis (2009 vs. 2007) was higher among individuals with a middle level of education (grades 7–12), but not in those with basic education (grades 1–6) and university. More specifically, sub-group analysis by sex revealed that increased risks of experiencing high stress levels were observed among females with middle and basic education during the crisis in 2009 as compared to the pre-crisis period in 2007 (but not with higher education level or males) [26]. Gili et al. also found that a college/graduate school level of education (reference groups: primary school/no schooling) was associated with a lower risk of having major depressive disorder during the crisis in Spain in 2010, while this association was not significant during the pre-crisis period (2006). Similarly, college/graduate school (as compared to those with primary school/no schooling) also had a lower risk of experiencing dysthymia, and this likelihood was higher during the economic crisis period (2010) than the pre-crisis period (2006) [1]. Those with complete high school education (as compared to primary school/no schooling) were also less likely to experience dysthymia during the crisis, but this association was not significant during the pre-crisis period [1]. Economou et al. found that a significant increase in the prevalence of major depression was observed among all education groups (<11 years, 12 years, >12 years) following the economic crisis in Greece when compared to the pre-crisis period (2008) [24].

#### Health behaviours

Gili et al. observed that higher education was negatively associated with alcohol abuse during the Spanish crisis in 2010 [1]. Similarly, Bor et al. found that during the US Great Recession in 2008–2009, those with a college degree were less likely to become frequent binge drinkers, as compared to those with less than a high school education [38]. Macy et al. also documented that having a higher education was associated with engaging in healthy behaviours (e.g. choosing food based on nutritional value, increased frequency of vigorous exercise, wearing a seatbelt and not smoking) both before and after the crisis but the associations generally weakened during the crisis [39]. McClure et al. demonstrated that women with middle or university level education (compared to basic) were more likely to quit smoking during the crisis (2009) as compared to the pre-crisis period (2007) [27]. Gili et al. also found that a higher level of education (compared to primary school/no schooling) was equally associated with a lower risk of alcohol abuse both before (2006) and during the economic crisis in Spain in 2010. Also, those with completed high school education (compared to primary school/no schooling) were less likely to abuse alcohol during the crisis period as compared to the pre-crisis period [1].

### Marital status and children in household

#### Physical health

Kondo et al. found that those who had never married (as compared to married) were more likely to report poor self-rated health following the Japanese economic recession (1991–2000) when compared to the pre-crisis period. In addition, the chances of experiencing poor self-rated health among divorced (as compared to married) people decreased in the post-crisis period as compared to the pre-crisis period. Those separated (as compared to married) were also more likely to experience poor health in the pre-crisis period, but after the crisis this difference was not observed [29].

#### Mental health

There was evidence from three studies of associations between marital status and stress levels [26], depression [24] and happiness [25] during economic crises. Hauksdottir et al. found that the probability of experiencing high stress levels during the crisis in Iceland (2009 vs. pre-crisis period in 2007) was greater among married and/or cohabiting persons but not in single/divorced or widowed. This held true for both genders, however, single/divorced females were also reported as being more likely to experience high stress levels during the crisis period (as compared to the pre-crisis period) [26]. Economou et al., in their study of the Greek economic crisis in 2011, observed a significant increase in the prevalence of major depression among single and married individuals, but not those widowed and divorced. During the crisis, married (as compared to single) had a higher chance of experiencing major depression [24]. Gudmundsdottir found that being married, committed in a relationship, or cohabiting, but not divorced or widowed (with being single as the reference group), was the strongest predictor of happiness both before (2007) and during the Icelandic economic crisis (2009) [25]. Hauksdottir et al. found that the chances of experiencing high stress levels during the Icelandic economic crisis (2009 versus pre-crisis period in 2007) were higher among persons with no children in the household, but not in households with one child or more. A gender-subgroup analysis confirmed this observation, but only for females without children [26].

#### Health behaviours

Bor et al. found that during the 2008–2009 period of the US Great Recession frequent binge drinkers were more likely to be unmarried [38]. Macy et al. observed that being married was associated with engaging in healthy behaviours (choosing food based on nutritional value, wear seatbelt and not smoke) before the US recession (2005). During the crisis period (2011), these associations strengthened, with the exception of seatbelt wearing where the association decreased, and new associations with exercising vigorously and checking food labels became significant [39]. Bor et al. also found that those with children aged <18 in the household were less likely to be frequent binge drinkers during the US Great Recession in 2008–2009 [38].

### Employment/Occupation

#### Physical health

Three studies investigated the effect of employment/occupation on physical health status [29,31,36]. Kim et al. examined changes in employment-related health inequalities according to occupational position among employed individuals in South Korea. The authors observed that the 1997 economic crisis was associated with a widening health gap in employment type related health inequalities in both professional and non-professional occupations, and for both genders. In addition, following the crisis the health gap continuously widened between female temporary/daily employees (limited contractual work of less than one year or daily contractual work of duration of one month or less) and those with permanent employment. A similar pattern was observed with daily male employees compared with permanently employed male employees [31]. In the study by Kondo et al. of the health impact of the Japanese economic recession (1991–2000), there was no association between occupational status and self-rated health among economically active people (managerial/administrative, professional, clerical/sales/service, manual, or any other paid job) before crisis. However, after the crisis, professionals, middle-class non-manual workers (clerical/sales/service), and other paid workers were more likely to report poor health compared to the highest class of workers (managerial/administrative). Unemployed and homemakers (when compared with the highest class workers, i.e. managerial/administrative) were more likely to report poor health both prior and following the crisis. More specifically, gender sub-group analysis showed that male middle-class non-manual workers (clerical/sales/service), as compared to high-class managerial/administrative workers, were more likely to report poor perceived health after economic crisis (1998–2001) as compared to the pre-crisis period (1986–1989). Similar findings apply to female homemakers and those unemployed in the post-crisis period. Among men, the unemployed were less likely to report poor health (compared to high-class managerial/administrative workers) during the post-crisis period as compared to the pre-crisis period [29]. Astell-Burt et al. investigated whether the prevalence of poor health at a population level increased concurrently with the rise in unemployment during the UK economic recession that started in 2008. During the crisis period (2008 and onwards) the likelihood of reporting poor self-rated health was higher among the unemployed compared to the employed, who also had a higher chance of having health problems such as respiratory and cardiovascular health problems (2009 and onwards) [36].

#### Mortality and suicide

Martikainen et al. examined the effects of unemployment on mortality following workplace downsizing and closure during the Finnish crisis of the early 1990s. The authors found that unemployed (as compared to employed individuals) were more likely to die during the crisis (1994), but the difference was less than in the pre-crisis period (1989). In addition, the crisis did not impact the association between unemployment and excess mortality among those who had been employed at workplaces undergoing large reductions in employment. Interestingly, mortality was lower among those who worked in a closed or substantially downsized establishment than among those working in establishments with a stable workforce size [21]. The study by Montgomery et al. of the Swedish crisis of the early 1990s examined the association between unemployment and all-cause mortality among men only. During the crisis (1990), unemployment was associated with an increased risk of all-cause mortality in people at all levels of cognitive function and education. This association was greatest among less well educated (compulsory education, up to 9 years) men and men with poor cognitive function, whereas after the crisis (2001) people with higher cognitive function and higher education were at greater relative risk of mortality following unemployment [34]. Wada et al. examined the impact of the Asian Crisis (1997–1999) on men in Japan by occupation and found that age-standardized mortality rates for the leading causes of death steadily decreased over the time (1980–2005) in all occupations, though managerial and professional workers had a lower risk of death in the pre-crisis period (compared to non-managerial and unemployed), but an increased risk of all-cause mortality during the economic crisis. The authors also found a rapid increase in suicides from the start of the Japanese crisis among all workers, with the greatest increase among management-level workers and professionals [30].

#### Mental health

There is evidence of an association between employment/occupation and general mental health status [2,23,33], stress levels [26], psychological distress [35], happiness [25] depression and mental illness [24,36], and sleep disturbances [37]. Rahmqvist et al., in a study of those aged 20–39 in the Swedish population, found the overall prevalence of psychological stress in the non-employed groups was consistently higher than in the employed group, throughout the studied period (1989–1995) in both genders. The authors also noted an increased likelihood of experiencing psychological stress (measured by GHQ) during the crisis of the early 1990s (1991–1995) as compared to the pre-crisis period (1989) in both genders (analysed separately). When limiting the analysis to employed individuals only, the increase in likelihood of experiencing psychological stress was retained [35].

Agudelo-Suarez et al. observed an increase in the prevalence of poor mental health between 2008 and 2011 (pre and during crisis periods) among employed and unemployed migrants of both genders, with particularly higher increase among unemployed of both genders. However, only in males did unemployment act as a significant predictor of poor mental health during the crisis (2011) when compared to the pre-crisis period (2009)[33]. Gili et al. also found that being unemployed (compared to employed) during the economic crisis in Spain (2010) carried even higher risk of experiencing both minor and major depressive disorders, generalized anxiety and multi-somatoform disorders, but not dysthymia or panic attack disorder, as compared to the pre-crisis period (2006). In addition, living in a household with unemployed family members also increased the risk of major depression during the crisis (2010) [1]. These findings were in line with those of Astell-Burt et al. who recorded how those unemployed (compared to employed) were more likely to report depression and poor mental health during the 2008 UK crisis (2009 and onwards) [36]. However, Katikireddi et al. observed that changes in employment status did not explain increases in poor mental health status in either males or females during two recessions recession in England (early 1990s and 2008 and onwards) [2]. Hauksdottir et al. found that employed individuals and students (but not unemployed, homemakers/being on parental leave, disabled or retired) had higher chances of experiencing high stress levels during the Icelandic economics crisis (2009) as compared to the pre-crisis period (2007). However, when the analysis was confined to women, a broader range of employment status (employed, students, being a homemaker/parental leave, and unemployed) were found to have an increased risk of experiencing high stress compared to the pre-crisis period. Occupational status was also analysed, with both non-skilled and skilled (but not executives) more likely to experience high stress levels during the crisis as compared to the pre-crisis period. In stratified analysis, occupation was important in predicting the experience of high stress levels during the crisis, as compared to the pre-crisis period, among non-skilled and no paid employment females. No significant association was observed for men [26]. Dregan et al. also found that, among individuals over the age of 50 in the UK, retirees were less likely to experience sleep loss through worry during the recession (1991), but not prior to the recession (1984–1985) [37]. Gudmundsdottir found that unemployment was associated with lower levels of happiness both before (2007) and after the 2008 Icelandic economic crisis (2009), and this association was stronger during the economic crisis [25]. Economou et al. found a significant increase in the prevalence of major depression in both employed and unemployed individuals during the Greek economic crisis (2011), as compared to the pre-crisis period (2008) [24].

#### Health behaviours

Four studies examined the role of employment/occupation on health behaviours [1,27,38,39]. Gili et al. found that during the Spanish economic crisis (2010) the unemployed (compared to employed) were at greater risk of alcohol abuse compared to the pre-crisis period (2006) [1]. Bor et al. also found that during the Great US Recession those who were unemployed for less than a year were more likely to be frequent binge drinkers whereas homemakers, students, retirees and those unable to work were less likely to be frequent binge drinkers (all compared to wage-employed) [38]. Macy et al. did not observe a significant association between change in work status or hours worked with health behaviours after adjustment in multivariate regression analysis [39]. In their study of the Iceland crisis, McClure et al. found that an individual’s employment status was not associated with their risk of having a smoking relapse, but retired women who had quit smoking at baseline (in 2007) were at increased risk of relapsing during the crisis, compared to employed women. Women not working because of disability, as compared to those employed, had also increased chances of quitting in 2009, as compared to 2007 [27].

### Income/financial constraints

#### Physical health and mortality

Lammintausta et al. analysed the effects of the Finnish Banking Crisis (1991–1995) on socioeconomic disparities in the incidence of coronary heart disease and corresponding mortality. The overall mortality and incidence rates, lower in females than males, were higher among low income tertiles in both genders (compared with middle and high income tertiles). The economic crisis did not affect the underlying downward trend in mortality and incidence rates among low and middle income tertiles in both genders, but the downward trend among those in the highest tertile slowed [20].

#### Mental health

There is evidence of associations between income/financial constraints, and general mental health status [2,23,29,33], stress levels [26], depression [1,24], sleep disturbances [37] and happiness [25]. Kondo et al.’s study of the Japanese economic recession (1991–2000) found that both females and males in the lowest income decile (as compared to the highest) were more likely to report poor mental health prior to the crisis than after the crisis (29).

The study on migrant workers in Spain between 2008 and 2011 recorded an increase in the prevalence of poor mental health status in those migrants of both genders with low salaries (below €1200 per month) but not those with monthly salaries above €1200. Low-earning males (but not females) were more likely to experience poor mental health during the crisis (2011) as compared to pre-crisis period (2008) [33]. Hauksdottir et al. found that the likelihood of experiencing high stress levels during the Icelandic crisis (2009 vs. pre-crisis period in 2007) was higher among female and males in the middle income bracket but not those with low or high income [26]. Additionally, financial hardship (as assessed by the index of personal economic distress) was found to be a predictor of major depression during the Greek crisis in 2011 [24]. Similarly, Gili et al. found that mortgage repayment difficulties and evictions were associated with elevated risk of major depression in Spain during economic crisis in 2010 [1]. In Iceland, financial difficulty (defined as “difficult making ends meet”) was negatively associated with happiness during the crisis (2009)[25]. Dregan et al. found that, among individuals over the age of 50 in the UK, financial problems over the last year appeared to be one of the major predictors of sleep disturbances during the 1991 recession but not in the pre-recession period (1984–1985)[37]. However, Katikireddi et al. found that adjustment for income did not explain changes in prevalence of poor general mental health status in either men or women during two economic financial recession in England (early 1990s and 2008 onwards) [2].

#### Health behaviours

Three studies examined the role of income/financial strain on health behaviours [27,38,39]. Bor et al. found that white Americans with household incomes over US$ 25,000 (compared to those earning <US$ 25,000) were more likely to engage in frequent binge drinking during the financial crisis of 2008–2009 [38]. Macy et al. found that reporting more financial strain was negatively associated with engaging in healthy behaviours (checking labels on purchased food, choose food based on nutritional value, frequent vigorous exercise, and seatbelt wearing and not smoking) before the crisis (2005) and there were even stronger negative associations during the crisis (2011). This negative association during the crisis period (as compared to pre-crisis period) was higher for checking labels on purchased food, choosing food based on nutritional value, frequently vigorously exercising, did not change for not smoking, and weakened for seatbelt wearing. The authors also found that men who moved from a lower income group (pre-crisis) to a higher income group (during the crisis) experienced an increased risk of smoking relapse, while men in the high income group before the crisis whose income dropped during the crisis had a decreased risk of relapsing [39]. McClure et al. found that male former smokers who moved into the high-income group in 2009 (as compared to 2007) experienced an increased risk of relapse (compared to those staying within lower income group in 2009), while those with high income at baseline (2007) whose incomes dropped (as compared to those with high income in 2009) had a decreased risk of relapsing [27].

### Personal beliefs

#### Mental health

There was evidence from two studies of an association between personal beliefs (including spiritual and political beliefs) and general mental health status [23] and happiness [28].

Viinamaki et al. found that lack of confidence about the future was a predictor of impaired mental well-being in both genders during and after the recession in 1990s in Finland. Its role as a predictor was particularly high at the onset of the economic crisis (1993) among males [23]. Doherty et al. observed positive associations with happiness of greater community trust, religious belief, and satisfaction with democracy, before and during the Irish economic crisis (2009). However, only the association between happiness and greater religious belief changed significantly during the crisis, with it increasing in 2009 compared with previous years [28].

### Health status

#### Mental health

There is evidence of associations between physical health status and general mental health status [23], happiness [25], and sleep loss [37]. Poor self-perceived physical health was a predictor of impaired mental health in females at the onset of the recession (1993) and during the recession and post-recession periods for males (1994–1995)[23]. Gudmundsdottir found that the association between better individual health status and happiness was stronger before (2007) than during the Icelandic economic crisis (2009) [25].

Dregan et al. found that health problems were the main predictor of sleep loss through worry (among individuals ages 50 and over) during both the pre-recession (1984–1985) and recession periods (1991) in the UK, but that this association was weaker during the recession period [37].

### Area of residence

#### Mental health

There is mixed evidence of associations between area of residence and general mental health status [2] and stress levels [26]. Katikireddi at al found no significant differences in the likelihood of poor mental health during two recessions in England (early 1990s and 2008 onwards) by area/level deprivation [2]. Whereas Hauksdottir found that the chances of experiencing high stress levels during the crisis (2009 versus pre-crisis period in 2007) were higher among females (but not males) residing in a city or village, but not in a farming community [26].

### Social relations

#### Mental health

There is evidence of associations between social relations and general mental health status [23,33] and happiness [25]. Agudelo-Suarez et al. observed an increase in the prevalence of poor mental health between 2008 and 2011 in migrants of both genders (with the highest increase among males) in Spain who reported having someone financially dependent in their countries of origin compared with those who did not. However, a higher chance of experiencing poor mental health during the crisis (2011), as compared to the pre-crisis period (2009), was seen only among males (and not females) with such relatives, and not in those without such dependents [33].

Viinamaki et al. found that both sexes suffered impaired mental health when experiencing problems with their partner in a relationship during the economic crisis. In the post-recession period, this remained important for women but became less important for men [23]. Gudmundsdottir found that more satisfactory social relationships with family members and friends were predictors of happiness both before (2007) and during the Icelandic crisis (2009), but the association became weaker during the crisis period [25].

## Discussion

The importance of understanding the characteristics of those who can withstand adversity has been recognised for many years, most notably by Antonovsky in his studies of Holocaust survivors [41]. However, work on such resilience has so far attracted little attention with respect to economic crises. Thus, to our knowledge, this is the first study to systematically review the evidence on risk and protective factors for health during economic crises. From the 22 studies that met our inclusion criteria, we identified 10 risk/protective factors associated with health during economic crises. These included age, gender, education, marital status and household size, employment/occupation, income/financial constraint, personal beliefs, health status, area of residence, and social relations.

It is not possible to ascertain from the identified literature whether those affected most were those who experienced the greatest shock or displayed the least resilience. An engineer, testing the resilience of a structure, would ascertain both the scale and nature of the shock to which it was exposed and the resulting response. However, the literature reviewed here only measures the net effect of shock and response. Consequently, the evidence offers some insight into vulnerability, which encompasses both the risk that an individual will be exposed to a shock (for example, by being in unstable employment or with low education) and the effect that the shock has on the individual, but less insight into the broader concept of resilience [42].

While recognising these limitations, the findings provide some insight onto factors influencing health outcomes and behaviours. Among the demographic factors, the results on the role of gender were ambiguous, although crises appeared to have a greater negative impact on mental health for women than men. This is somewhat surprising given the extensive evidence that men tend to be more likely to complete suicides during crises [9,43] and raises questions about the validity of the methods used, with a number of possible biases arising, such as response, survivorship bias, and social desirability. However, as noted above, what is missing is the potentially differential scale of the shock experienced. Thus, within Europe the consequences of job loss vary for men and women depending on the type of welfare state [44].

There was no consistent pattern according to age. Again, it is conceivable that the economic impact of crises on different ages will vary, reflecting different policy choices, such as whether governments protect pensions or invest in job creation schemes, which will benefit those of working ages. The impact of marital status also varied, which is again surprising given the body of previous evidence that unmarried men are especially vulnerable during political and economic difficulties [45].

Among the socio-economic risk-factors, lower income levels were associated with greater vulnerability to outcomes such as cardiovascular disease, overall mortality and poor mental health. In general, those with more precarious employment were more vulnerable to mental ill health. The association with education was mixed, although higher education seemed to be protective.

The most commonly examined health outcomes were mental health (general mental health, stress, depression) and changes in (un)healthy behaviours, followed by physical health and mortality (including attempted and completed suicides). These outcomes were likely chosen since most studies covered a short period of time that precluded measurement of longer-term changes in outcomes.

The methodological quality of the studies was generally good, with most of the studies rated as ‘strong’ and having a ‘high quality’ of reporting (low or moderate risk of bias). However, all of the studies included in this review examined one of three possible combinations: pre- and post-crisis; pre-crisis and during crisis; or during crisis and post-crisis periods. A longitudinal study design capturing all three periods would be more appropriate to examine the changing role of a specific factor as the crisis unfolds. This reinforces the case for establishing long-term monitoring systems, ideally with panel data, such as the Russian Longitudinal Monitoring Survey and the German Socioeconomic Panel [46,47].

A greater variety of analytical methods could also be employed to examine the causal relationships between risk and/or protective factors and outcomes. For example, methods such as dynamic modelling or structural equation modelling could be useful to examine underlying relationship between factors and causality. The use of multilevel modelling could better understand community- and macro-level influences on health outcomes.

More conceptually, future studies are required that expand the approach to understanding health risks, vulnerability and resilience during financial crises. Few studies adopted a comprehensive approach to a wide range of factors potentially associated with health outcomes, and there is some evidence that the impact of the financial crisis cannot always be adequately explained by conventionally accepted demographic or socio-economic factors [40]. Future studies should therefore go beyond the standard demographic and socioeconomic factors typically included in the papers in this review, and examine other potential factors, such as elements of social capital such as social relations and networks, sources of support, and trust, which have been shown to influence health in other settings [48,49,50]. As noted above, research is also required to understand community- and macro-level influences on health. Importantly, such work should incorporate the more comprehensive concept of resilience.

Another related limitation was the lack of reference in the studies to any theoretical framework used to inform study design. Inclusion of such frameworks could help to understand the phenomenon under study. For example, while a number of potentially appropriate frameworks exist on vulnerability and resilience which could be extremely valuable in the context of financial crises [13,15,51], empirical research has yet to apply or test such frameworks.

This review is subject to certain limitations. One is restricting the search to English language articles. Another is the exclusion of grey literature, but a preliminary search found no grey literature meeting the inclusion criteria. The review also employed narrative analysis, rather than meta-analysis, but this was due to the heterogeneity of study designs, outcomes, exposures, and risk and protective factors. It should also be acknowledged that even in an economy not experiencing a defined recession, a large number of people can experience individual economic shocks such as losing jobs or having homes repossessed. Conversely, a large proportion of people in an economy experiencing recession may not experience any fall in living standards. Such nuances are important, but were outside the scope of the paper. It should also be recognised that many developing countries have very low standards of living. So, even if they do not experience “economic crises” in the definitional sense of the word, and their economies may in fact be growing at fast rates, the people living there will have much lower standards of living than people living in the west during the crisis years. This review would not have included information on these populations.

## Conclusions

In this systematic review we examined the factors that influence resilience to negative health outcomes and behaviours during economic crises. Given the limitations in the volume and design of the available literature, and thus the mixed nature of the evidence, it provides a first step in disentangling some of the risk and protective factors that may be of importance in economic crises and thus may inform health policies and activities to support more vulnerable populations. However, substantially more research is required to effectively address vulnerability and help build resilience.

## Supporting Information

S1 Table Search strategy. (DOCX)

S2 Table Quality assessment. (DOCX)

S1 PRISMA Checklist PRISMA Checklist. (DOC)

S1 Quality Appraisal Tool (DOCX)

S1 Fig Quality assessment. (TIF)
