# Supplementary material for: A Systematic Review on Health Resilience to Economic Crises
Source: PLoS One. 2015 Apr 23;10(4):e0123117. doi: 10.1371/journal.pone.0123117 (PMC4408106; doi:10.1371/journal.pone.0123117)
Supplement: S1 Table — (DOCX) [file pone.0123117.s001.docx]

# Search strategy

| Objective | To examine evidence on associations between risk and/or protective factors and health during economic crises |
| --- | --- |
| Literature type | Published peer-reviewed articles |
| Study design | Primary quantitative longitudinal (panel/cohort and repeated cross-sectional) containing: (i) exposure to a specific economic crises; (ii) changes in health outcomes/behaviours over time; and (iii) statistical data on associations of risk/protective factors with health outcomes |
| Study settings | Global |
| Key definitions/terms | Economic crisis: We consider economic crisis as a recession and use the National Bureau of Economic Research (NBER) definition of recession as “a significant decline in economic activity spread across the economy, lasting more than a few months, normally visible in real GDP, real income, employment, industrial production, and wholesale-retail sales. A recession begins just after the economy reaches a peak of activity and ends as economy reaches its trough” (17).  Risk and/or protective factor. We consider a risk factor any attribute, characteristic or exposure of an individual that increases the likelihood of developing a disease or injury (16) . Examples of possible risk/ and/or protective factors of relevance to this study include demographic factors (e.g. gender, age, social-economic factors (e.g. employment, income, education, social relations), lifestyle (e.g. safe sex, tobacco and alcohol consumption), and environmental factors (e.g. living conditions). |
| Inclusion Criteria | Primary quantitative longitudinal (panel/cohort and repeated cross-sectional) containing: : (i) exposure of economic crises; and (ii) changes in health outcomes and/or harmful health behaviours over time; and (iii) statistical data on risk and/or protective factors. |
| Exclusion Criteria | General health effects of poverty, unemployment without a direct link to economic crises/ shocks/recession  Qualitative studies  Descriptive studies of trends in health outcomes during economic crises which do not provide statistical data on individual risk/protective factors.  Studies reporting on outcomes of health care utilisation (i.e. not on health behaviours or outcomes).  Studies on impact of economic crises on health system performance |
| Time period | No date restrictions |
| Databases | EMBASE, Global Health, MEDLINE, PsycINFO, Scopus, Web of Knowledge (Web of Science) will be searched using a combination of free text and subject heading terms by title and abstract |
| Languages | English only |
| Search period | Searches will be performed between July and August 2013 |

**Database search terms**

| **Database** | **TEXT / Abst. / keyword** | **Limits/restrictions** |
| --- | --- | --- |
| EMBASE | economic shock OR economic recession OR recession OR economic crisis OR financial crisis OR fiscal crisis OR banking crisis OR economic depression OR economic hardship OR economic insecurity OR austerity OR financial constraint OR economic downturn OR economic change OR economic breakdown OR economic turmoil OR economic stagnation OR economic adversity  OR economic turbulence OR macroeconomic fluctuation  OR  MeSH: exp. Economic recession.sh. | human |
| MEDLINE | economic shock OR economic recession OR recession OR economic crisis OR financial crisis OR fiscal crisis OR banking crisis OR economic depression OR economic hardship OR economic insecurity OR austerity OR financial constraint OR economic downturn OR economic change OR economic breakdown OR economic turmoil OR economic stagnation OR economic adversity  Or economic turbulence OR macroeconomic fluctuation  OR  MeSH: exp. Economic recession/exp gross domestic product.sh. | human  journal articles |
| Global Health | economic shock OR economic recession OR recession OR economic crisis OR financial crisis OR fiscal crisis OR banking crisis OR economic depression OR economic hardship OR economic insecurity OR austerity OR financial constraint OR economic downturn OR economic change OR economic breakdown OR economic turmoil OR economic stagnation OR economic adversity  OR economic turbulence OR macroeconomic fluctuation  OR  MeSH (economic depression or economic crises).sh. | journal articles |
| PsycINFO | economic shock OR economic recession OR recession OR economic crisis OR financial crisis OR fiscal crisis OR banking crisis OR economic depression OR economic hardship OR economic insecurity OR austerity OR financial constraint OR economic downturn OR economic change OR economic breakdown OR economic turmoil OR economic stagnation OR economic adversity  OR economic turbulence OR macroeconomic fluctuation  OR  MeSH exp Financial Strain/sh. | human |
| Scopus | “economic shock” OR “economic recession” OR “recession” OR “economic crisis” OR “financial crisis” OR “fiscal crisis” OR “banking crisis” OR “economic depression” OR “economic hardship” OR “economic insecurity” OR “austerity” OR “financial constraint” OR “economic downturn” OR “economic change” OR “economic breakdown” OR “economic turmoil” OR “economic stagnation” OR “economic adversity” OR economic turbulence OR macroeconomic fluctuation | **Sources:**  Social science and medicine, world development, international journal of health services, lancet, plos one, international journal of social economics  **Document type:**  Articles  Reviews |
| Web of  knowledge | economic shock OR economic recession OR recession OR economic crisis OR financial crisis OR fiscal crisis OR banking crisis OR economic depression OR economic hardship OR economic insecurity OR austerity OR financial constraint OR economic downturn OR economic change OR economic breakdown OR economic turmoil OR economic stagnation OR economic adversity  OR economic turbulence OR macroeconomic fluctuation | **Selected research areas:**  Sociology, health care sciences services, social sciences, behavioural sciences, social issues, demography, social work, family studies  **Document types:**  articles |
