# Supplementary material for: A Systematic Review on Health Resilience to Economic Crises
Source: PLoS One. 2015 Apr 23;10(4):e0123117. doi: 10.1371/journal.pone.0123117 (PMC4408106; doi:10.1371/journal.pone.0123117)
Supplement: S2 Table — (DOCX) [file pone.0123117.s002.docx]

# Quality assessment of the included studies

| **Authors** | **Year** | **Country** | **Study design*** | **Representa-tiveness I*** | **Representa-tiveness II*** | **Confounding*** | **Data collection*** | **Data analysis*** | **Reporting*** | **Overall rating*** |
| --- | --- | --- | --- | --- | --- | --- | --- | --- | --- | --- |
| Agudelo-Suarez et al. | 2013 | Spain | **2** | **2** | **2** | **2** | **3** | **1** | **1** | **2** |
| Astell-Burt et al. | 2013 | UK | **2** | **1** | **N/A** | **1** | **2** | **1** | **1** | **1** |
| Bor et al. | 2013 | USA | **2** | **1** | **N/A** | **1** | **2** | **1** | **1** | **1** |
| Doherty et al. | 2013 | Ireland | **2** | **1** | **N/A** | **1** | **2** | **1** | **1** | **1** |
| Dregan | 2009 | United Kingdom | **2** | **1** | **4** | **2** | **2** | **1** | **1** | **2** |
| Economou et al. | 2013 | Greece | **2** | **1** | **1** | **1** | **1** | **1** | **1** | **1** |
| Gili et al. | 2012 | Spain | **2** | **1** | **N/A** | **1** | **1** | **1** | **1** | **1** |
| Gudmundsdottir et al. | 2013 | Iceland | **2** | **1** | **2** | **1** | **3** | **1** | **1** | **2** |
| Hauksdottir et al. | 2013 | Iceland | **2** | **1** | **2** | **1** | **2** | **1** | **1** | **2** |
| Katikireddi et al. | 2012 | England | **2** | **1** | **4** | **1** | **3** | **1** | **1** | **2** |
| Kim et al. | 2003 | South Korea | **2** | **1** | **3** | **2** | **2** | **1** | **1** | **2** |
| Kondo et al. | 2008 | Japan | **2** | **1** | **N/A** | **1** | **2** | **1** | **1** | **1** |
| Lammintausta et al. | 2012 | Finland | **2** | **1** | **N/A** | **1** | **2** | **1** | **1** | **1** |
| Lee et al. | 2009 | South Korea | **2** | **1** | **N/A** | **2** | **2** | **1** | **2** | **2** |
| Macy et al. | 2013 | USA | **2** | **1** | **1** | **1** | **2** | **1** | **1** | **1** |
| Martikainen et al. | 2007 | Finland | **2** | **1** | **N/A** | **2** | **3** | **1** | **1** | **2** |
| McClure et al. | 2012 | Iceland | **2** | **1** | **N/A** | **1** | **2** | **1** | **1** | **1** |
| Montgomery et al. | 2013 | Sweden | **2** | **1** | **1** | **1** | **2** | **1** | **1** | **1** |
| Ostamo et al. | 2001 | Finland | **2** | **1** | **N/A** | **2** | **2** | **2** | **1** | **2** |
| Rahmqvist et al. | 1998 | Sweden | **2** | **1** | **N/A** | **2** | **2** | **1** | **1** | **2** |
| Viinamaki et al. | 2000 | Finland | **2** | **1** | **N/A** | **1** | **2** | **1** | **1** | **1** |
| Wada et al. | 2012 | Japan | **2** | **1** | **N/A** | **2** | **2** | **1** | **1** | **2** |
| NB. Scores for risk-of-bias range from 1 (low risk of bias; high methodological quality) to 3 (high risk of bias; low methodological quality). Score 4 stands for no rating.  * Study design refers to the distinction between experimental and observational, and cross-sectional and longitudinal studies. Representativeness refers to the extent to which  the study population is generalizable to the population it was drawn from. Representativeness II refers to representativeness due to withdrawals and drop-outs. Confounding refers to the adjustment for relevant confounding factors in analyses. Data collection refers to the extent in which valid and reliable instruments were used for data collection. Data analysis refers to the methods used for data analysis (for example only descriptive analyses versus more complex analyses). Data reporting refers to the extent in which authors were specific in their reporting about hypotheses and probability value. | | | | | | | | | | |
