# Supplementary material for: A Systematic Review on Health Resilience to Economic Crises
Source: PLoS One. 2015 Apr 23;10(4):e0123117. doi: 10.1371/journal.pone.0123117 (PMC4408106; doi:10.1371/journal.pone.0123117)
Supplement: S1 Quality Appraisal Tool — (DOCX) [file pone.0123117.s004.docx]

# Quality Appraisal Tool

**STUDY DESIGN**

(**Q1**) The study design is:

1. Experimental
   - 1. Individual-randomised
     2. Group-randomised
     3. Non-randomised
2. Observational
   - 1. Cross-sectional
     2. Longitudinal (also natural experiment or pre-post tests)
     3. Case-control
3. Any other method or did not state method (i.e. pre-post test without control group)

(**Q2**) Was this an intervention study?

**Yes** – proceed

**No** – go to question 7

(**Q3**) Is the intervention of interest clearly described?

1. Yes
2. No

(**Q4**) Were (groups of) subjects randomized into intervention groups?

1. Yes
2. No
3. Not applicable

(**Q5**) Was the intervention assignment concealed from participants and care givers until recruitment was completed?

1. Yes
2. No
3. Can’t tell

(**Q6**) Was (were) the intervention or exposure status of participants concealed from the outcome assessors?

1. Yes
2. No
3. Can’t tell

(**Q7**) Were power/sample size calculations conducted?

1. Yes, details of calculation provided
2. Yes, no details provided
3. Not reported or post hoc computation
4. Not applicable (using an existing database and referring to design article*

**Rating study design: Strong:** Q1 is 1

**Moderate:** Q1 is 2

**Weak:** Q1 is 3

**Rating blinding: Strong:** Q5 and Q6 are 1

**Moderate:** Q5 or Q6 is 1**; or** Q5 or Q6 are 3

**Weak:** Q5 and Q6 are 2; **or** Q5 and Q6 are 3

**(no rate is given when study is not an intervention study)**

*• If the study is using data from a large existing database such as HSE, NHANES, BRFSS etc, often the authors refer to the design paper of the original study and no information in the present article is being described about power calculations, validity of tools et.*

**REPRESENTATIVENESS (selection bias)**

(**Q8**) Is the spectrum of individuals selected to participate likely to be representative of the wider population who experience the intervention/exposure/situation?

1. Very likely
2. Somewhat likely
3. Not likely (selected group of users e.g., volunteers)
4. Can´t tell (no information provided)
5. Not applicable (using an existing database and authors refer to design article)

(**Q9**) What percentage of the selected participants agreed to participate?

1. ⁮ …………..%
2. ⁮ Can’t tell
3. ⁮ Not applicable

(**Q10**) Were inclusion/exclusion criteria specified and number of exclusions reported?

1. Criteria and number of exclusions reported
2. Criteria or number of exclusions not reported
3. Criteria and number not reported

**Rating: Strong:** Q8 is 1

**Moderate:** Q8 is 2

**Weak:** Q8 is 3 or 4

**No rating:** Q8 is 5

**REPRESENTATIVENESS (withdrawals and drop-outs)**

(**Q11**) Were withdrawals and drop-outs reported in terms of numbers and reasons per group?

1. Numbers and reasons provided
2. Numbers but no reasons provided
3. Can’t tell (if longitudinal data)
4. Not applicable (if cross-sectional data or if using an existing database and authors refer to design article)

*If Q11 is 1 or 2, proceed to Q12. Otherwise, proceed to Q13.*

(**Q12**) What was the loss to follow-up/percentage completing the study? (If % differs by groups, record the lowest)

1. ⁮ …………..%
2. ⁮ Not provided
3. ⁮ Not applicable

**Rating: Strong:** Q11 is 1

**Moderate:** Q11 is 2

**Weak:** Q11 is 3

**No rating:** Q11 is 4

**CONFOUNDERS**

(**Q13#**) What confounders were the analyses adjusted for?

…………………………………………………………………………………………………...

…………………………………………………………………………………………………...

(**Q13**) Were analyses appropriately adjusted for confounders?

1. For most confounders
2. For some confounders
3. No or can’t tell

*The following are examples of confounders: race, sex, marital status/family, age, SES (income or class), education, health status, pre-intervention score on outcome measure.*

*Considering the study design, were appropriate methods for controlling confounding variables and limiting potential biases used? Confounding can be addressed by appropriate use of randomization, restriction, matching, stratification, or multivariable methods. Sometimes use of a single method may be inadequate. Some biases can be limited by institution of data collection or study procedures that support validity of the study (e.g. training and/or blinding of interviewers or observers, interviewers and observers are different from interventions’ implementers etc). Example: if between-group differences persist after randomization or matching, statistical control should also have been used.*

**Rating: Strong:** Q13 is 1

**Moderate:** Q13 is 2

**Weak:** Q13 is 3

**DATA COLLECTION**

(**Q14**) Were validity, reliability or appropriateness of the data collection tools discussed?

- 1. Both validity and reliability were discussed
  2. a. Validity or reliability were discussed

b. A national dataset was used and authors provided adequate information to find information on validity and reliability

- 1. None of them were discussed

**Rating: Strong:** Q14 is 1

**Moderate:** Q14 is 2

**Weak:** Q14 is 3

**DATA ANALYSIS**

(**Q15**) Were appropriate statistical analyses conducted (including correction for multiple tests where applicable)?

1. a. Statistical methods were described and were appropriate and comprehensive – sophisticated approach

b. Statistical methods were described and were appropriate and comprehensive –simple approach

1. Statistical methods were described and less appropriate
2. No description of statistical methods or inappropriate methods

**Rating: Strong:** Q15 is 1

**Moderate:** Q15 is 2

**Weak:** Q15 is 3

**REPORTING**

(**Q16**) Are the hypothesis/aim/objective of the study clearly described?

1. Yes
2. No

(**Q17**) Are the main outcomes to be measured clearly described?

1. Yes
2. No

(**Q18**) Are the main findings clearly described?

1. Yes
2. No

(**Q19**) Have actual probability values been reported

*(i.e. p=0.345 instead of p>0.05; same goes for t-values, 95%CIs etc)?*

1. Yes
2. No

**Rating: Strong:** Q16 and Q19 are 1

**Moderate:** Q16 or Q19 are 1

**Weak:** Q16 and Q19 are 2

Studies can have between six and eight component ratings. The overall rating for each study is determined by assessing the component ratings.

If seven ratings have been given;

**Strong** will be attributed to those with no WEAK ratings and at least four STRONG ratings;

**Moderate** will be given to those with one WEAK rating or fewer than four STRONG ratings;

**Weak** will be attributed to those with two or more WEAK ratings.

If six ratings have been given;

**Strong** will be attributed to those with no WEAK ratings and at least three STRONG ratings; **Moderate** will be given to those with one WEAK rating or fewer than three STRONG ratings;

**Weak** will be attributed to those with two or more WEAK ratings.

If five ratings have been given;

**Strong** will be attributed to those with no WEAK ratings and at least two STRONG ratings;

**Moderate** will be given to those with one WEAK rating or fewer than two STRONG ratings;

**Weak** will be attributed to those with two or more WEAK ratings.

If four ratings have been given;

**Strong** will be attributed to those with no WEAK ratings and at least two STRONG ratings;

**Moderate** will be given to those with one WEAK rating or fewer than two STRONG ratings;

**Weak** will be attributed to those with two or more WEAK ratings.

The final decision of both reviewers will be: **strong, moderate, or weak**.
